# Eye regression in blind *Astyanax* cavefish may facilitate the evolution of an adaptive behavior and its sensory receptors

**DOI:** 10.1186/1741-7007-11-81

**Published:** 2013-07-11

**Authors:** Richard Borowsky

**Affiliations:** 1 Department of Biology, New York University, Washington Square, New York, NY 10003, USA

**Keywords:** *Astyanax*, Regressive evolution, Eye loss, Cavefish, QTL, Antagonistic pleiotropy, VAB

## Abstract

The forces driving the evolutionary loss or simplification of traits such as vision and pigmentation in cave animals are still debated. Three alternative hypotheses are direct selection against the trait, genetic drift, and indirect selection due to antagonistic pleiotropy. Recent work establishes that *Astyanax* cavefish exhibit vibration attraction behavior (VAB), a presumed behavioral adaptation to finding food in the dark not exhibited by surface fish. Genetic analysis revealed two regions in the genome with **quantitative trait loci** (QTL) for both VAB and eye size. These observations were interpreted as genetic evidence that selection for VAB indirectly drove eye regression through antagonistic pleiotropy and, further, that this is a general mechanism to account for regressive evolution. These conclusions are unsupported by the data; the analysis fails to establish pleiotropy and ignores the numerous other QTL that map to, and potentially interact, in the same regions. It is likely that all three forces drive evolutionary change. We will be able to distinguish among them in individual cases only when we have identified the causative alleles and characterized their effects.

## 

Cave *Astyanax* are attracted to a vibrating needle at the water’s surface while eyed surface individuals are not [1]. This “vibration attraction behavior” (VAB) likely facilitates the detection of food in the dark cave and may be an important behavioral adaptation to cave life. Jeffery and colleagues recently reported the mapping of two unlinked quantitative trait loci (QTL) for VAB in a hybrid cross between surface fish and cave fish from the Pachón population. They showed that VAB is mediated by a small number of superficial neuromasts (“SN EO” or “SN” herein) in the eyeless orbit of the cave fish which are absent from the orbits of surface fish [2]. They demonstrated the existence of two QTL controlling proliferation of SN, each co-mapping with one of the VAB QTL. Presumably, the VAB and SN QTL in each cluster are manifestations of single underlying genes or features, detectable through their effects on both structure and function.

The authors also demonstrated QTL for eye size differences in the same two clusters and found that the Bayesian credible intervals for all three QTL in each cluster overlapped. The authors interpreted the close linkage of the VAB/SN and eye size QTL as evidence that the traits are functionally and evolutionarily related. In both clusters, cave alleles for VAB/SN cause increases in sensitivity and greater numbers of neuromasts, presumably adaptive changes, while cave alleles for eye size cause smaller eyes. The authors concluded 1) that all three traits reflect the pleiotropic effects of single genes or closely clustered genes, 2) that positive selection for VAB most likely drove the regression of eye size by antagonistic pleiotropy or through hitchhiking, and 3) that this may be a general mechanism to account for eye regression in cave animals.

Unfortunately, the conclusions are unsupported by the data and may be incorrect for several reasons. First, while the three QTL are presented as if they were the only traits in each cluster, in fact, both clusters are in genomic regions already known to be crowded with QTL. The cluster on linkage group (LG)2 aligns with a well-documented hotspot for cave adaptation (Figure 1A), which contains QTL for eye size, condition factor, rate of weight loss on fasting, number of melanophores, number of branched anal fin rays, depth of the caudal peduncle, number of SO3 bones in the skull, and number of maxillary teeth [3]. The allelic substitution data for the QTL (Table 1) show that cave alleles for some of the traits are adaptive and for others are maladaptive. Thus, there are numerous potential interactions in this region, none of which is individually strongly supported on the sole basis of the proximity of QTL. The hypothesis may or may not be true, but we will not know until the genes are identified and characterized.

**Figure 1:**
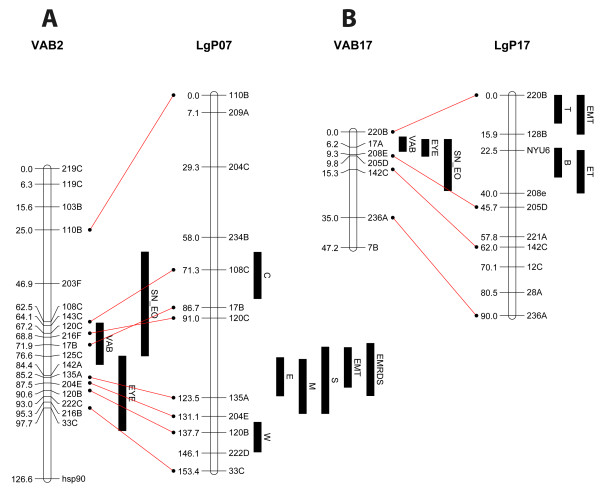
**QTL mapped in a cross between surface and Pachón cave fish cluster in the genome.** Vibration attraction behavior (VAB)2 and VAB17 are redrawn from Figure Three of Yoshizawa *et al.*[2]. These are aligned with the homologous linkage groups LgP07 and LgP17 from Protas *et al.*[3]. The black bars show the locations of QTL mapped in the crosses. QTL traits are as follow: C, condition factor; D, depth of the caudal peduncle; Eye or E, eye size; M, number of melanophores; R, number of branched anal fin rays; S, number of SO3 bones; SN EO, superficial neuromasts in the eyeless orbit; T, number of maxillary teeth; VAB, vibration attraction behavior; W, rate of weight loss on fast. QTL mapped on LgP07 and LgP17 are from [3] and [6]. Methods for mapping QTL and for detection of multi-trait QTL are given in [3]. EMRDS is a multi-trait QTL with significant correlation to variability in Eye size, numbers of Melanophores, number of anal fin Rays, Depth of the caudal peduncle, and number of So3 bones. Similarly, EMT is a multitrait QTL with significant contributions for Eye size, numbers of Melanophores and numbers of maxillary Teeth.

**Table 1:** **Trait values for the QTL on LgP07 and LgP17 (Figure **1**)**

					**Evolutionary**
	**Trait**	**Genotypic classes**			**significance**
		**CC**	**CS**	**SS**	
LgP07					
	Eye size	0.82	0.98	1.03	Adaptive (?)
	Melanophore #	50.2	32.9	23.4	Neutral
	Maxillary teeth	2.50	2.60	2.68	Maladaptive
	Condition factor	1.05	0.98	0.96	Adaptive
	Weight loss	−0.42	−0.34	−0.33	Maladaptive
	Anal fin rays	22.2	22.2	21.8	Neutral (?)
	Depth caudal ped.	0.102	0.102	0.098	Maladaptive (?)
	Suborbital (SO3) width	0.67	0.63	0.59	Neutral (?)
LgP17					
	Eye size (ET)	0.96	0.90	0.81	Maladaptive (?)
	Eye size (EMT)	1.16	1.11	0.98	Maladaptive (?)
	Melanophore # (EMT)	40.36	26.74	25.3	Neutral
	Maxillary teeth	3.07	2.44	2.11	Adaptive
	Maxillary teeth (ET)	3.99	3.29	3.08	Adaptive
	Maxillary teeth (EMT)	3.12	2.23	1.90	Adaptive
	Thoracic ribs	11.8	12.3	13.1	Neutral (?)

*CC* cave allele homozygote, *CS* heterozygote, *SS* surface allele homozygote Trait values for LgP07 were taken from [3]. Trait values for multi-trait QTL were calculated from allelic substitution values in [3] or computed from the original files used for QTL detection [6].

The other cluster on LG17 also aligns with a region previously identified as having QTL for eye size, number of maxillary teeth, number of melanophores and number of ribs (Figure 1B). Thus, as with LG2, there is no evidence that the associations of VAB, SN and eye size QTL reflect any exclusive interactions among them. Interestingly, the eye QTL previously identified in this region has the opposite polarity of almost all known eye QTL [4], including the one identified by Jeffery and colleagues [2], because the cave fish homozygotes have larger eyes than the surface fish homozygotes (Table 1). The differences in polarity suggest that the two eye QTL are caused by different alleles at the same or closely linked loci, which is not surprising because they were discovered in different mapping progenies. Nevertheless, the contrasting polarities highlight the dangers in making sweeping generalizations based on few observations.

Second, the argument that selection for VAB drove eye regression hinges on there being significant alignment of QTL for the two traits. But in LG2, LOD the (logarithm of the odds) score profiles of the QTL for VAB and eye size do not coincide. The peaks for the two QTL are over 20 cM apart (Figure Three B in [2]), and it is highly unlikely that the two traits reflect pleiotropic effects of the same gene, a point already commented upon [5], or even that linkage is tight enough for effective hitchhiking. Overlap of Bayesian credible intervals provides little information about the probability that the positions of two QTL coincide; the less well defined the QTL, the greater the overlap.

Third, most QTL for eye size in *Astyanax* have negative polarity, with the cave homozygote classes [6]. This observation is consistent with direct selection against eyes. It is inconsistent, however, with genetic drift or indirect selection through pleiotropy, both of which predict QTL with mixed polarities. Finally, and importantly, it is difficult to envision any evolutionary series consistent with the hypothesis that selection for VAB drove eye regression. VAB is mediated by superficial neuromasts in the eyeless orbit, which structure did not exist in the ancestral state.

In summary, there is no genetic support for the assertion [2] that selection for VAB drove the loss of eyes in *Astyanax* cavefish, much less the suggestion that it is a general mechanism. Based on all the current evidence, the most parsimonious explanation is that eye regression provided a clear field for the proliferation of superficial neuromasts and perhaps passively facilitated the evolution of VAB.

### Abbreviations

LG: linkage group; QTL: quantitative trait loci; SN EO: superficial neuromasts in the eyeless orbit; VAB: vibration attraction behavior.

### Competing interests

The author declare that he has no competing interests.
